# Basic self-disorders may play a role in the development of depression in schizophrenia: a seven-year follow-up study

**DOI:** 10.3389/fpsyt.2024.1521366

**Published:** 2025-01-08

**Authors:** Elisabeth Haug, Merete Glenne Øie, Ingrid Hartveit Svendsen, Paul Møller, Barnaby Nelson, Ingrid Melle

**Affiliations:** ^1^ District Psychiatric Centre Gjøvik, Division of Mental Health Care, Innlandet Hospital Trust, Brumunddal, Norway; ^2^ Department of Research, Innlandet Hospital Trust, Brumunddal, Norway; ^3^ Department of Psychology, University of Oslo, Oslo, Norway; ^4^ Department of competence and education, Innlandet Hospital Trust, Brumunddal, Norway; ^5^ Department of Health Sciences, Norwegian University of Science and Technology Gjøvik, Gjøvik, Norway; ^6^ Department of Mental Health Research and Development, Division of Mental Health and Addiction, Vestre Viken Hospital Trust, Drammen, Norway; ^7^ Centre for Youth Mental Health, University of Melbourne, Parkville, VIC, Australia; ^8^ Orygen, Parkville, VIC, Australia; ^9^ Adult Psychiatry Department, Institute of Clinical Medicine, University of Oslo, Oslo, Norway; ^10^ Section for Clinical Psychosis Research, Oslo University Hospital, Oslo, Norway

**Keywords:** schizophrenia, depression, self-disorders, psychosis, follow-up,

## Abstract

**Introduction:**

Depression is the most common co-morbid psychiatric disorder in patients with schizophrenia and has a negative effect on functional outcomes and quality of life. There are several possible pathways leading to depressive symptoms in schizophrenia. Self-disorders are disturbances in the deepest, pre-reflective level of the self and are suggested to be core features of schizophrenia. The relationship between self-disorders, and depressive symptoms has only been studied to a limited extent, and there are no longitudinal studies. This study aimed to explore the relationship between self-disorders at baseline and the development of depression over the follow-up period

**Methods:**

Self-disorders were examined with the Examination of Anomalous Self-Experience (EASE) instrument in 31 patients with schizophrenia during their first treatment. Seven years later we measured the total number of depressive episodes since the start of treatment.

**Results:**

We found a clear association between self-disorders at baseline and the total number of depressive episodes over the follow-up period.

**Discussion:**

Self-disorders may play a role in the development of depression in schizophrenia. This may have implications for therapeutic approaches targeting a condition that is characterized by considerable suffering and an elevated risk of suicide.

## Introduction

Depression is the most common co-morbid psychiatric disorder in patients with schizophrenia, affecting almost one-third of stabilized patients (1), and is characterized by considerable suffering and an elevated risk of suicide.

Depression has a negative effect on functional outcomes and quality of life in patients with schizophrenia (2, 3). There are several possible pathways leading to depressive symptoms in schizophrenia (4, 5). Depression could be a core dimension of psychosis in line with the negative symptoms (6). It may also be a psychological reaction to being diagnosed with schizophrenia, the social consequences of the disease, or a reaction to psychotic relapse (4, 7, 8).

In a recent review and meta-analysis, the prevalence of depression in schizophrenia was not associated with positive psychotic symptoms, negative symptoms, or stable remission (1), leading the authors to suggest that depression in schizophrenia is a co-morbid disorder due to overlapping risk factors rather than directly linked to the disorder itself.

Despite intense research, schizophrenia, and its underlying mechanisms are only partly understood. The current definitions of schizophrenia are descriptive and a-theoretical. The definitions’ validity appears reduced by not incorporating the complex subjective phenomena that many see as central to the disorder (9, 10). Basic self-disorders (SDs) are suggested to be core features of schizophrenia, both from a theoretical viewpoint and based on empirical findings (11–15). SDs are disturbances in the basic sense of self, e.g., the person’s sense of being the subject of their own experiences, and refer to disturbances of the deepest, pre-reflective level of the self, affecting cognition and self-awareness (16). SDs include certain subtle forms of depersonalization, anomalous experiences of cognition and stream of consciousness, self-alienation, pervasive difficulties in grasping familiar and taken-for-granted meanings, unusual bodily feelings, and existential reorientation (17). SDs in schizophrenia are associated with positive symptoms, first-rank symptoms, negative symptoms, disorganised symptoms, and formal thought disorders (18–22). Low levels of SDs at the start of the first treatment also predict increased chances of recovery in psychotic disorders (23).

The relationship between SDs and depressive symptoms has, however, only been studied to a limited extent. One study found a significant association between basic symptoms and depression in patients with schizophrenia, using the Bonn Scale for the Assessment of Basic Symptoms, which measures anomalous subjective experiences (‘basic symptoms’) related to SDs (24). The baseline assessment of the current study indicated that SDs in schizophrenia were associated with depression and suicidality, mediated by the level of self-esteem (25, 26). SDs are considered relatively stable traits in schizophrenia (27), while depression is episodic. However, in the current follow-up study SDs were less stable than expected, and there was a significantly lower level of SDs at follow-up (28).

The main hypothesis would be that SDs contribute to the development of depression, either directly or through increasing other risk factors for depression. However, previous studies of this issue have been cross-sectional, and thus cannot say anything directly about the direction of association. For example, it could be that depressed patients report more SDs because they are more sensitive to these phenomena. As far as we know, there have been no longitudinal studies examining the relationship between SDs and depression in schizophrenia. The current paper reports on a seven-year follow-up of a representative cohort of first-episode psychosis patients with baseline and follow-up measures of SDs and a comprehensive clinical assessment battery, including measures of depression. The main aim of this study was to explore the relationship between SDs at baseline and the development of depression over the follow-up period. A greater understanding of the association between SDs and depression may have implications for therapeutic approaches targeting a condition that is characterized by considerable suffering and an elevated risk of suicide.

## Material and methods

### Participants

The current study is a seven-year follow-up study of a representative cohort of first-episode DSM-IV schizophrenia patients from a geographical catchment area in Norway, including both urban and rural areas (**Table 1**). A total of 35 patients (61%) from the initial cohort participated, and data on the total number of depressive episodes were available for 31 of these patients (54% of the original sample). See Svendsen and colleagues for a description of the study (28).

**Table 1:** Clinical and sociodemographic variables.

	Baseline	Follow-up
Male sex (%)	51,6	
Age (Median, Range)	25,8 (17-48)	
SDs (Mean, SD)	23,3 9,9	15,3 9,7
CDSS (Median, Range)	8 (0-22)	3 (0-20)
PANSS (Mean, SD)	75,6 17,1	54,5 13,2
Total number of depressive episodes, (Median, Range)		1 (0-22)

### Assessments

#### Assessment of self-disturbances

At baseline and follow-up, SDs were assessed with the *Examination of Anomalous Self Experience* (EASE) manual (17). The EASE manual consists of five domains: (1) Cognition and stream of consciousness, (2) Self-awareness and presence, (3) Bodily experiences, (4) Demarcation/transitivism, and (5) Existential reorientation. The EASE manual usually aims to capture the lifetime experiences of SDs, and therefore at baseline, we registered lifetime experiences. To measure any change in SDs at follow-up, we rated SDs experiences during the last two years. For a detailed description of this assessment, see Svendsen and colleagues (28). There is considerable overlap between single items and domains, and both items and domains are statistically highly inter-correlated (18, 29, 30). We have thus used the total EASE score in the analyses.

#### Clinical assessments

At follow-up, we measured the total number of depressive episodes since the start of treatment, using a semi-structured interview with a year-by-year timeline, using the diagnostic criteria for a major depressive episode to identify them. This information was cross-checked with information from the medical charts. Since depression is episodic, we considered this variable more robust than the level of depression at follow-up for investigating the effect of SDs on the development of depression Assessment of the level of depression at baseline and follow-up was based on the Calgary Depression Scale for Schizophrenia (CDSS) (31). The CDSS is the most widely used scale for assessing depression in schizophrenia and discriminating depression from negative and positive symptoms of the disorder (32). Symptom severity at baseline was assessed using the Structured Clinical Interview for the Positive and Negative Syndrome Scale (SCI-PANSS) (33).

### Statistical analyses

All analyses were performed with the statistical package SPSS, version 29.0 (SPSS inc. Chicago, IL, USA). We used descriptive statistics to characterise the sample Mean and standard deviations, or median and range, are reported for continuous variables and percentages for categorical variables. The total number of depressive episodes over the follow-up period had a markedly skewed distribution, thus a logarithm transformation of the variable was performed and used in all subsequent analyses. We used the z-scores for SD and PANSS at baseline. The relationship between SDs and CDSS at baseline was investigated using Pearson correlation, and the relationship between SDs and CDSS at follow-up was investigated using Spearman`s rho because CDSS at follow-up was not normally distributed. We used Spearman`s rho to investigate the potential relationship between the EASE-domains and the total number of depressive episodes. We used multiple linear regression analyses to measure the association between SD at baseline and the total number of depressive episodes (log10 of the number + 1) over the follow-up period, after controlling for the influence of symptoms as measured by the PANSS total score at baseline. The variables were entered hierarchically in steps with PANSS at the first step and EASE at the second. The model fit was evaluated as good based on histograms and residual plots.

### Ethics

All participants provided consent at baseline to be contacted again for the follow-up. The study was approved by the regional Committee for Medical Research Ethics and the Norwegian Data Inspectorate.

## Results

Clinical and sociodemographic variables are shown in **Table 1**. The age of the patients was skewed, with the main part of participants in their early twenties. We found that SDs at baseline had a significant positive association with the total number of depressive episodes over the follow-up period, which was maintained after correcting for symptom severity (PANSS) at baseline (**Table 2**). We also found significant positive associations between SDs at baseline and the severity of depression (CDSS) at baseline (r=0,45, p = 0,01) and between SDs at follow-up and the severity of depression (CDSS) at follow-up (r=0,58, p<0,001). The five EASE domains are highly intercorrelated however no single domain was statistically significantly associated with the total number of depressive episodes.

**Table 2:** Multiple linear regression analysis with total number of depressive episodes as the dependent variable and SDs at baseline as independent variable, controlling for PANSS at baseline.

Variable	Rsq change	B	Std Error	Beta	t	p	95% CI for p	
Step 1	0,051							
PANSS total at baseline		-.006	.005	-.227	-1.253	.220	-.016	.004
Step 2	0,147							
PANSS total at baseline		-0,006	0.004	-0,232	-1,371	0,181	-0,150	0,003
EASE total at baseline		0,017	0,008	0,384	2,268	0,031	0,02	0,033

Model F=5,142, Sig F=0,031, adj Rsq=0,141.

## Discussion

Our main finding is a clear association between SDs at baseline and the total number of depressive episodes over the follow-up period, indicating that SDs may play a role in the development of depression in schizophrenia. We also found positive associations between SDs and the level of depression both at baseline and at follow-up.

This is in line with earlier cross-sectional studies that have shown an association between SDs and depression (24–26).

SDs include loss of the sense of owning one’s own experiences, thoughts, and actions, and also disturbances in the basic self to the extent that the person has a feeling of not existing or not being human. In addition, SDs include the feeling that the world is insecure, not real or that they do not belong to our shared reality. This may contribute to depressive symptoms. As an example: During the EASE interview one person said he was convinced that he was dead because he experienced that other people did not interact with him. Another person described a pervasive feeling of loneliness based on the experience that neither she nor the rest of the world, including other people existed. One felt homeless in our shared world because he felt like an alien and thought he belonged to another planet. Some said they felt inhuman, like a robot, and some thought that other people were robots as well, which contributed to a feeling of loneliness and insecurity. A lot of them said they experienced a pervasive anxiety, like something really bad was to happen, and they felt very vulnerable and exposed.

A recent study has highlighted the significance of so-called pseudo neurotic symptoms in schizophrenia, which include depressive symptoms as well as anxiety and obsessive-compulsive symptoms (34). This study has found the symptoms to be cross-sectionally and longitudinally associated with SD and to predict low level of functioning prospectively. Such symptoms appear to be relatively stable and integrated with the core pathology of schizophrenia, rather than being secondary or comorbid phenomena. Our findings on the association between SD and depressive episodes support this understanding, suggesting that depressive symptoms in schizophrenia may reflect deeper disruptions in the sense of self.

SDs may consist of primary and secondary factors (35). Some features (possibly secondary features) may be expressions of an underlying general “P” factor (36). while other features (possibly the primary features) may be more specific to the schizophrenia spectrum. Further research is required to test this hypothesis. The knowledge of the possible association between SDs and depression may have implications for phenomenological psychotherapy for patients with schizophrenia through improving clinicians’ understanding of the lived experience of these patients (37).

### Strengths

We included patients consecutively from all treatment facilities in a large combined rural/urban catchment area, and the number of male and female participants was almost identical, ensuring a high degree of representativity in the study population.

### Limitations

The study sample is small, increasing the risks of both type I and type II errors. Additionally, there might be some recall bias in the participants’ reports of depressive episodes, even though these reports were cross-checked with medical charts. While our findings suggest a potential link between self-disorders (SDs) and the development of depression in schizophrenia, the results are correlational in nature. Further research is needed to establish a causal relationship.

## Data Availability

The raw data supporting the conclusions of this article will be made available by the authors, without undue reservation.
